# Microtubules and Lis-1/NudE/Dynein Regulate Invasive Cell-on-Cell Migration in Drosophila

**DOI:** 10.1371/journal.pone.0040632

**Published:** 2012-07-13

**Authors:** Nachen Yang, Mikiko Inaki, Adam Cliffe, Pernille Rørth

**Affiliations:** 1 Institute of Molecular and Cell Biology, Singapore, Singapore; 2 Department of Biological Sciences, The National University of Singapore, Singapore, Singapore; Stockholm University, Sweden

## Abstract

The environment through which cells migrate in vivo differs considerably from the in vitro environment where cell migration is often studied. In vivo many cells migrate in crowded and complex 3-dimensional tissues and may use other cells as the substratum on which they move. This includes neurons, glia and their progenitors in the brain. Here we use a Drosophila model of invasive, collective migration in a cellular environment to investigate the roles of microtubules and microtubule regulators in this type of cell movement. Border cells are of epithelial origin and have no visible microtubule organizing center (MTOC). Interestingly, microtubule plus-end growth was biased away from the leading edge. General perturbation of the microtubule cytoskeleton and analysis by live imaging showed that microtubules in both the migrating cells and the substrate cells affect movement. Also, whole-tissue and cell autonomous deletion of the microtubule regulator Stathmin had distinct effects. A screen of 67 genes encoding microtubule interacting proteins uncovered cell autonomous requirements for Lis-1, NudE and Dynein in border cell migration. Net cluster migration was decreased, with initiation of migration and formation of dominant front cell protrusion being most dramatically affected. Organization of cells within the cluster and localization of cell-cell adhesion molecules were also abnormal. Given the established role of Lis-1 in migrating neurons, this could indicate a general role of Lis-1/NudE, Dynein and microtubules, in cell-on-cell migration. Spatial regulation of cell-cell adhesion may be a common theme, consistent with observing both cell autonomous and non-autonomous requirements in both systems.

## Introduction

Eukaryotic cell migration has been studied very effectively in simplified cell culture models. It is usually an actin driven progress involving actin-dependent cellular protrusions and force for both traction and forward propulsion derived from actin/myosin contractility [1]. Active cell migration also requires cell polarization, a difference between the front and the back of the cell [2], which may be directed by external cues (guidance). Compared to the actin cytoskeleton, the role of the microtubule cytoskeleton in cell migration is less fixed. Microtubules can be critical for front versus back polarity and directionality [3], [4]. However, there are also migratory cell types in which microtubules suppress cell polarity [5], [6]. Actively dividing cells including tissue culture cells usually have a prominent microtubule organizing center (MTOC) associated with the centrosome, which orients growth of microtubules with plus ends generally extending outwards, toward the cell periphery. Additional signaling can lead to added bias such that microtubule plus ends are most clearly enriched at the leading edge or front of the cell [7], as observed in multiple cell types. The bias in polarity of the microtubule cytoskeleton may direct vesicle transport or nuclear movement, influence focal adhesions and interact with the actin cytoskeleton. Overall, it appears that even in the simplified cell culture situation, migrating cells can make use of polarized microtubules in multiple ways, depending on the cell type or type of movement.

For understanding the roles and regulation of cell migration in health and disease, it is critical to determine how cells migrate under normal circumstances, in their respective tissues. This is technically difficult, as the 3-dimensional deep tissues generally do not allow as sensitive and detailed imaging as the simple 2-dimensional cell culture systems. Some features of cell migration are likely similar in vivo and in vitro, but some are not, in particular when considering cells that migrate on, and squeeze between, other cells. One interesting class of such cell-on-cell migration is neuronal migration in the brain [8], [9], including the movement of neural precursors out of the ventricular zone. The microtubule cytoskeleton appears to play an important role in neuronal migration. Mammalian Lis-1 was originally identified as a dosage sensitive gene that could cause lissencephaly, a severe developmental disease of the brain characterized by mislocalization of cortical neurons [10]. Further analyses have confirmed the roles of both Lis-1 and interacting proteins including Dynein in neuronal migration [11]. Mutations in the tubulin alpha gene, encoding one of the two microtubule subunits, also cause lissencephaly [12] and related brain abnormalities are seen in beta tubulin mutants [13], reinforcing the importance of the microtubule cytoskeleton in this context. In addition to considering the potentially different substrate features in 3-D tissues and 2-D dishes, some types of cell migration in vivo are collective [14], [15]. In collective migration, cells migrate together and influence one another while doing so. This influence can be via robust, but generally dynamic, physical association between the migrating cells (as in sprouting angiogenesis) or regulatory interactions (as in neural crest cells). Considering the interaction with substrate cells as well, this means that, at any point in time, more than one type of cell-cell interaction may be important for a migrating cell in the tissue.

**Figure 1:**
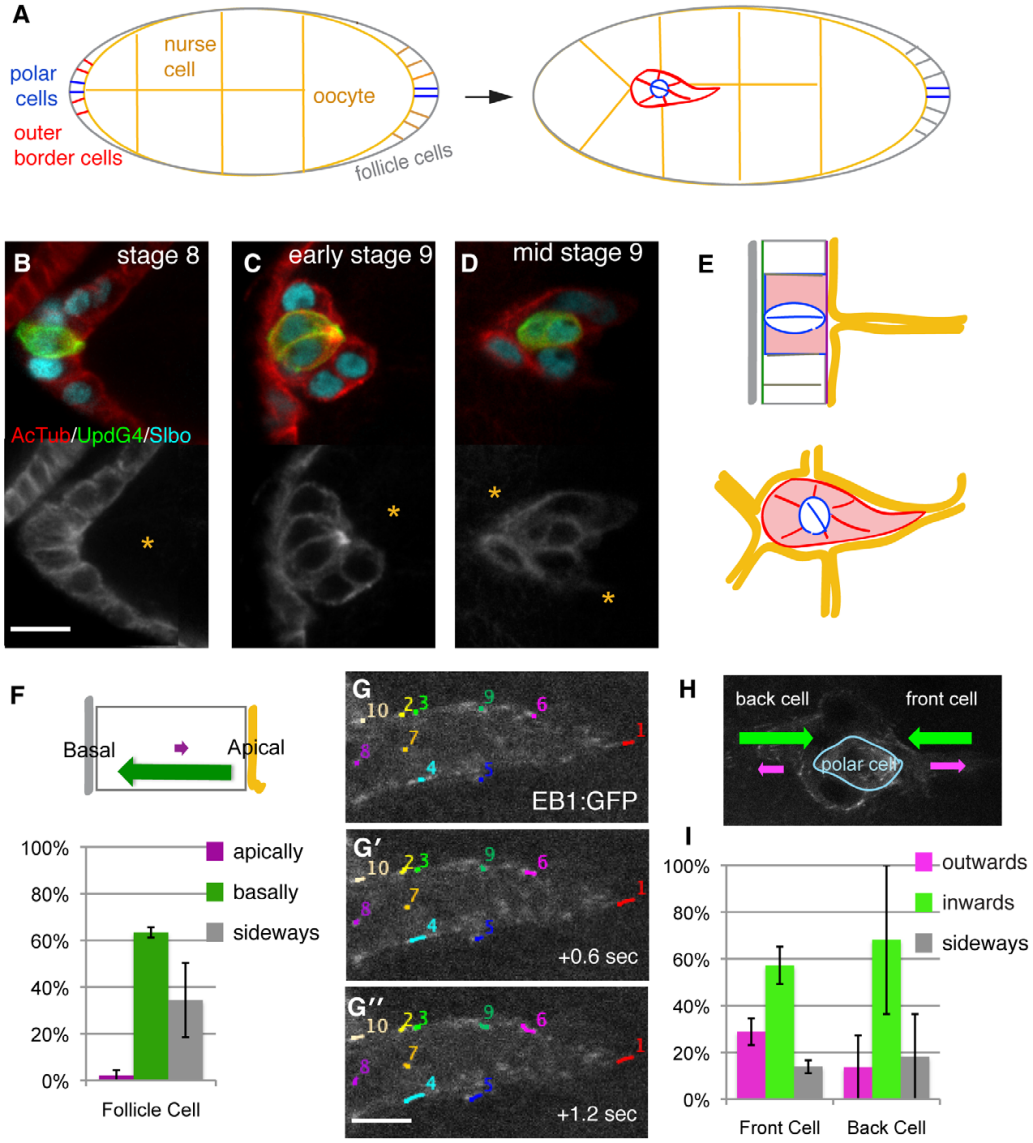
Microtubule organization and polarity in border cell clusters. (A) Schematic of egg chambers before (left) and after (right) initiation of border cells migration. In this and all other figures, anterior is to the left and cells migrate to the right. Border cells (red) and polar cells (blue) are indicated. (B-D) Close-up of border cell clusters at indicated stages (before, at and after initiation of migration) from genotype: Upd-Gal4; UAS-PH-GFP (green, identifies polar cells) stained with anti-slbo (cyan) and anti-actylated tubulin (red above and white below). (Scale bar: 10 µm). Yellow asterisk indicates nurse cells. (E) Schematics illustrating cell organization before (upper panel) and during (lower) border cell migration. Apical surface (purple line) is toward yellow germ line cells. (F) Direction of tracked EB1-GFP dots in follicle cells showing microtubule growth bias. Schematic above, quantification below; 22 tracks from 2 movies. (G) Three cut-out frames from movie S1 illustrating tracking of EB1-GFP dots in the front part of the front border cell (Scale bar: 4 µm). (H) Summary of direction of EB1-GFP dots in migrating border cells showing microtubule growth bias. (I) Quantification of EB1-GFP dots in border cell clusters; schematic above, quantification below. 131 tracks from 7 movies were analyzed, SEM indicated; P<0.05 for outwards versus inwards movement.

The migration of border cells in the Drosophila ovary has been established as a model to study guided and collective cell migration in vivo [16]. The border cell cluster is a group of about 8 cells that are directly derived from the anterior follicular epithelium of an egg chamber (Fig. 1A). The cells do not divide, but undergo transcriptionally induced changes in cell shape and behavior to become migratory [17], [18]. Border cells form a tight group and invade the underlying germ line tissue to migrate to the posteriorly localized oocyte. The homophilic cell-cell adhesion molecule DE-cadherin is absolutely required both in the migratory cells and in the germ line cells for migration [19], [20], suggesting DE-cadherin is responsible for substrate traction. Which adhesion molecules are responsible for keeping cells of the cluster together has not been clearly determined. Live analyses of border cell migration show a very dynamic cluster behavior [21], [22]. In 3D, each cell migrates with a speed of about 1.5 micron/minute but the net forward movement of the cluster varies depending on cluster behavior [23], with large cellular extensions from the front cell associated with more efficient forward motion. Net forward movement of the cluster is dependent on input from the guidance receptors PVR (PDGF/VEGF receptor related) and EGFR [21], [22], [23]. Genetic analysis and imaging of border cell movement indicates it is, as expected, an actin dependent process [24], [25] [21], [26]. However, little is known about the potential role of the microtubule cytoskeleton in this collective, cell-on-cell migration.

## Results

### Investigating the Roles of Microtubules in Border Cell Migration

To start investigating the possible roles of the microtubule cytoskeleton in border cell migration, we first determined the distribution of stable and dynamic microtubules at different stages of the process. We focused on initiation of migration and during the first half of their movement to the oocyte as this allowed reasonable imaging quality in the live imaging approaches below. The 2 interior cells of the forming border cell cluster, the anterior polar cells (blue outlines in Fig. 1A) are specialized signaling cells. Polar cells are present at the anterior and posterior of every egg chamber throughout oogenesis. At the earliest stage of border cell cluster formation, stage 8, the outer cells of the cluster (henceforward called border cells) start expressing differentiation markers such as Slbo (Fig. 1B) but they are still part of the follicular epithelium. Detection of stable, acetylated microtubules showed a discreet apical accumulation in the polar cells in an MTOC-like structure, in agreement with recent published observations [27]. This well-organized microtubule cytoskeleton has a unique function in polar cells [27]. There was no observable MTOC in either border cells or in follicle cells. The border cells display loosely organized bundles of stable microtubules enriched at the cell cortex both when they initiate migration (Fig. 1C) and during their active migration (Fig. 1D). These are visible when compared to the surrounding germ-line cells (orange asterisks) that have few stable microtubules. Thus border cells retain a microtubule cytoskeleton that is superficially similar to that of the follicular epithelium from which they derive.

To visualize dynamic microtubules and their polarity in border cells, we analyzed egg chambers from females with transgenic expression of EB1-GFP, a marker for growing microtubule plus ends. To track the fast-moving EB1-GFP comets, live imaging was done in one focal plane, which allowed short-term tracking. Most follicle cells form a simple epithelium with a well-defined apical to basal polarity and, as for other epithelial cells [28], the microtubule plus ends are enriched basally [29]. As expected, there was a strong bias for EB1-GFP comets moving basally in these cells (Fig. 1F). In border cells, EB1-GFP marked comets were also observed (see movie S1). Figure 1G shows EB1-GFP over time in the front half of a cell initiating migration. The overall bias in the front cell was for EB1-GFP comets to move away from the front edge, both when the cluster was initiating migration and during migration (Fig. 1G–I). In the back cell of a cluster, the comets more often moved away from the back (Fig. 1I). The preferential inward movement of comets in both front and back cells indicates that microtubules are organized mainly with respect to cell organization within the cluster, rather than the direction of movement of the cluster (Fig. 1H). Outer membranes of the migratory border cells retain some apical characteristics [19] (Fig. 1E), so this bias is similar to that of follicle cells. The bias was less strict, however, with some EB1-GFP moving in the opposite direction, indicating some reorganization of microtubules occurs upon formation of the motile cluster. When considering the leading migratory border cell, microtubule growth bias is essentially opposite from what has been observed in many individually migrating cells.

**Figure 2:**
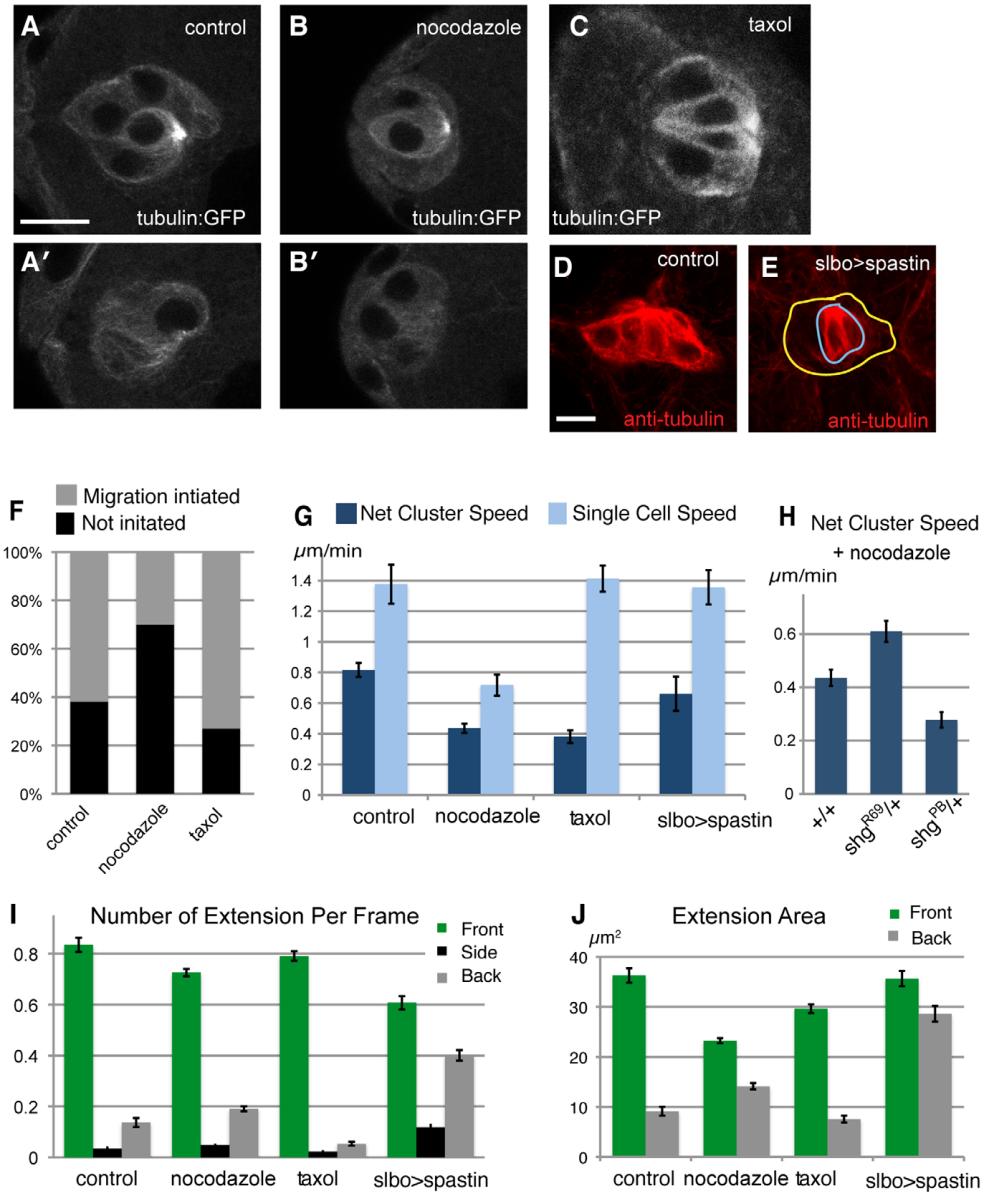
Probing the role of microtubules in border cells migration with drugs. (A–C). Microtubules visualized by tubulin-GFP fusion. For A and B, two focal planes are shown, top includes the polar cells, bottom only border cells. (D, E). Microtubules visualized in fixed samples by anti-tubulin staining (red) in control (D) and slbo>Spastin (E) females, showing strong reduction in migratory border cells (yellow outline), not polar cells (blue outline). Scale bars 10 µm. (F) Percent successful initiation of migration during movies (up to 2 hours) of early stage 9 egg chambers; n = 21 to 50. Significant difference (P<0.05) for nocodazole versus control. (G) Quantification of net cluster migration and of tracked single cell speeds from live analysis (see movies S2 and S3); n = 8 to 21; SEM indicated. Differences for net migration (drugs versus control) and nocodazole versus control in single cell speed are significant (P<0.05). (H) Nocodazole treatment of wild type versus females heterozygous for mutation in shg (encodes DE-cadherin); R69 is a null allele, PB4354 specifically disrupts border cell expression [53]. (I) Presence of cellular extensions from the migrating cluster (per movie frame) divided into front side and back quadrant relative to direction to oocyte. Most differences to control, including nocodazole front and back, taxol back, are significant (P<0.001). (J). Average area of front and back extensions from migrating clusters. Back extensions differ significantly from control for both nocodazole and spastin, (P<0.001). For drug treatments, egg chambers are treated with DMSO vehicle alone (control), or with 2 µM nocodazole or 2 µM taxol from the onset of imaging. Genotypes (except H): slboGal4/tubulin-GFP (A-C) or slboGal4, 10×GFP/+ (F-J) for control and drug treatments; slboGal4/UAS-spastin-GFP for slbo>spastin.

The immediate effects of perturbing the microtubule cytoskeleton can be assessed using specific drugs that interfere with microtubule dynamics. To monitor the efficacy of the drugs in this whole-tissue system, we visualized stable and dynamic microtubules by expression of tubulin-GFP under control of a promoter that drives expression in all cells of the tissue (Fig. 2A). Drug concentrations were titrated to not cause perturbations of the egg chamber integrity and nurse cell nuclear dynamics. Under these conditions, the de-polymerizing drug nocodazole caused attenuation of the strong focus of microtubules in polar cells (upper panel in Fig. 2A and 2B), and marked reduction of the cortical microtubules in border cells (bottom panels). Treatment with taxol, which stabilizes microtubules, caused microtubules in the border cells become denser throughout the cytoplasm (Fig. 2C). More dramatically, the normally very dynamic microtubules in the surrounding germ line cells were also visualized. These changes were observed within minutes of drug addition, allowing immediate live assessment of effects of perturbing the microtubule cytoskeleton.

To analyze the effects of drug treatments on border cell migration, we followed the process in real time (as described in [22], [23] see movies S2 and S3). For egg chambers in which border cell migration had not yet started at the onset of imaging and drug addition, we monitored the ability of clusters to initiate migration within a set time (Fig. 2F). For clusters that had initiated migration, we monitored both net cluster movement and manually tracked individual cells within the cluster to assess their basic cell motility (Fig. 2G). Nocodazole treatment caused an impairment of all three features, whereas taxol treatment reduced net cluster speed, leaving the other features normal. For comparison, incubation with cytochalasin D, a potent inhibitor of actin polymerization, caused a complete block of border cell migration (see movie S4), confirming that this is an actin-dependent process. To gain more insight into the effects that perturbations of microtubules had on cell movement, we quantified the frequency (Fig. 2I) and size (Fig. 2J) of extensions. Extensions are defined by an automated procedure as protrusions that exceed a minimum size and emanate from the “body” of the migrating cluster [23]. They are classified according to direction: front is toward the oocyte (front quadrant), back in the opposite direction and side covers the rest. Front extensions come from front cells, back extensions come from rear cells, and both represent active protrusions. Front extensions enhance net cluster forward movement; and a strong reduction in these would be expected to decrease net cluster movement significantly [23]. We observed some differences in drug-treated samples, such as an increase in size and abundance of back cell extensions upon nocodazole treatment. But a clear bias for front extensions was generally retained after drug treatment, indicating appropriate perception of guidance cues. Net forward cluster movement requires directionality and invasiveness as border cells invade into the germ-line tissue. Overall, instantaneous perturbation of microtubules caused defects in border cell migration that appeared to indicate reduced cell motility and invasiveness.

The drug experiments presented above do not distinguish autonomous and non-autonomous effects. Border cells migrate upon other cells, the giant nurse cells. In principle, microtubules could be important either in the migrating cells, in the substrate cells, or both. In order to disrupt microtubules in a cell-specific way, we induced expression of the microtubule severing protein Spastin [30] in migratory border cells using a specific expression inducer (slbo-Gal4). Variable microtubules reduction was observed prior to migration, so initiation was not analyzed. An obvious reduction of microtubules was observed in migrating border cells (Fig. 2D, E). However, this caused no significant perturbation of net cluster migration or single cell speed (Fig. 2G). Thus the immediate effect of nocodazole on border cell movement could be due to perturbation of microtubules in the nurse cells. In support of this idea, the migration defect caused by nocodazole treatment was suppressed by reduction of DE-cadherin levels in the tissue (shg^R69^/+ in Fig 2H), whereas reduction in the border cells only (shg^PB^/+) had the opposite effect. Migrating spastin-expressing border cells did show some abnormal features, however. The strong bias for front extensions being both more prevalent (Fig. 2I) and larger (Fig. 2J) than extensions in other directions was reduced. In particular, extensions from back cells were more abundant and larger. We have previously shown that, under control of guidance signaling from PVR and EGFR, extensions from back cells differ from front cell extensions not only in size but also in being “non-productive”, that is, they do not affect net cluster movement [23]. Non-productive extensions from back cells could explain why net forward movement of spastin-expressing clusters was normal, rather than the front and back cells engaging in a tug of war with limited net movement as observed for guidance defective clusters. In summary, microtubules appear to function in both migrating cells and the substrate cells to allow effective migration but have different effects in each compartment.

### The Role of Stathmin in Border Cell Migration

**Figure 3:**
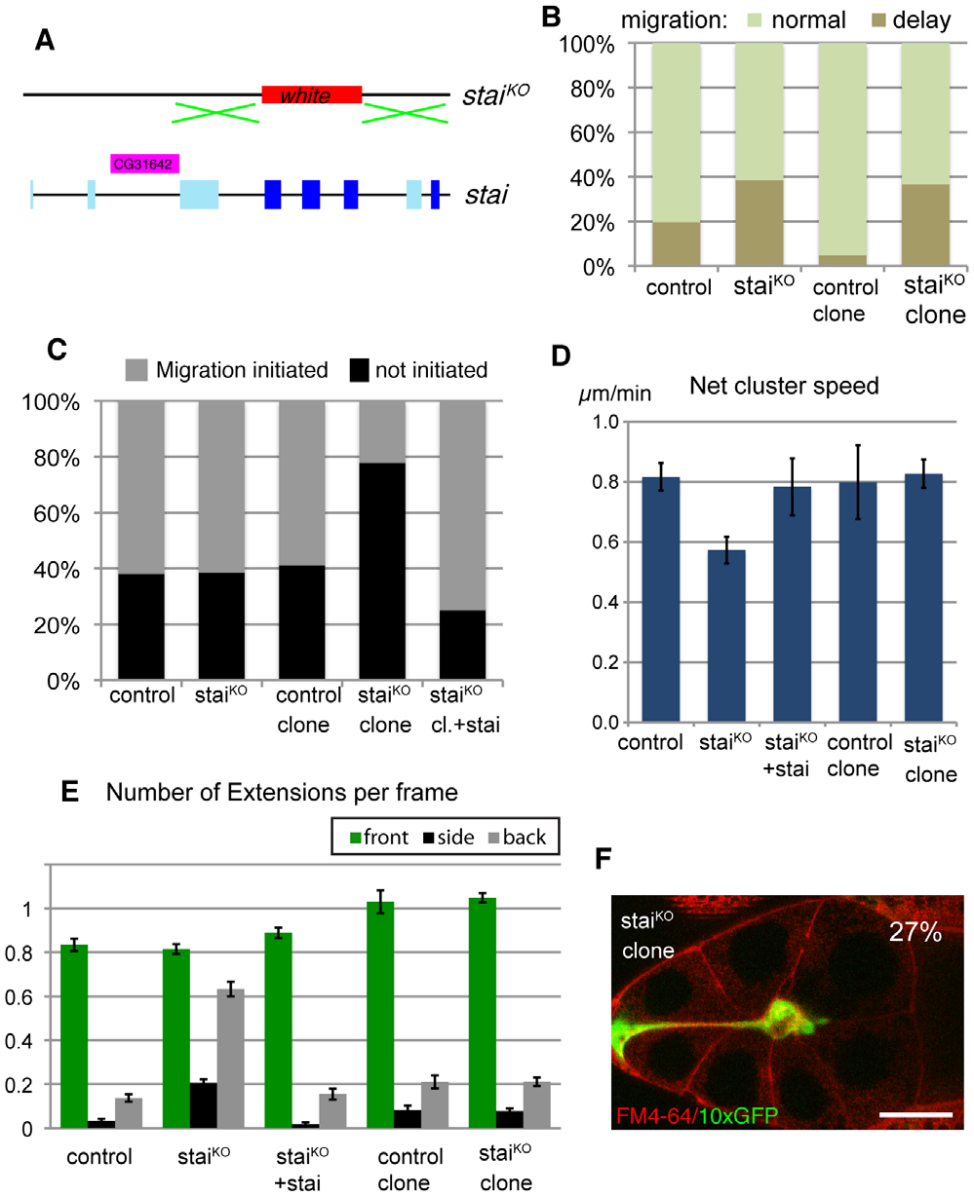
The role of Stathmin in border cell migration. (A) Schematic of the Drosophila stathmin (stai) locus and the “knock-out” stai^KO^ construct, replacing most of the coding region with the white marker gene. Conserved coding exons used in all stathmin isoforms shown in dark blue, alternative coding exons shown in light blue. (B) Quantification border cell migration status for mid/late stage 9 egg chambers. Genotypes: slboGal4,UAS-10xGFP/+ (control); stai^KO^/stai^KO^; slboGal4,UAS-10×GFP/+ (stai^KO^); GFP positive border cells from hsFLP/+;FRT40/FRT40,Gal80;slboGal4/UAS-10×GFP (control clone) and hsFLP/+;stai^KO^,FRT40/FRT40,Gal80;slboGal4/UAS-10×GFP (stai^KO^clone). n = 26–219; Elevated delay frequency in stai^KO^clones is significant (P<0.001). (C) Percent successful initiation of migration during movies (up to 2 hours) of early stage 9 egg chambers. Genotypes as in B; +stai indicates presence of tub-stathmin transgene driving ubiquitous stathmin expression or UAS-stai in stai^KO^ clone. n>13 per genotype; the defect in stai^KO^ clone is significant (P<0.05). (D) Quantification of net migrating speed from movies of border cell clusters. Genotypes are as indicated in B and C. The difference between stai^KO^ and control is significant (P<0.05). (E) Presence of cellular extensions from the migrating cluster (per movie frame), difference between stai^KO^ and control is significant for back and side (P<0.001). (F) Snapshot from a movie of GFP-positive (green) stai^KO^ border cell clone; a few cells that were adjacent to the border cells are also GFP positive. The back cell does not detach in the full 2-hour movie. FM4-64 (red) marks membranes. Scale bar: 40 µm.

Genetic analysis could help identify specific microtubule regulators and effectors important in this context and reveal in which compartment they are needed. Stathmin is a conserved regulator of microtubules that promotes depolymerization of microtubules and can itself be negatively regulated by multiple phosphorylation events [31], [32], [33]. We had previously found Stathmin to be upregulated in border cells in a Slbo dependent manner, suggesting a possible role in these cells [17]. Our initial phenotypic analysis of Stathmin function was incorrect, however, as the severe defects observed were due to disruption of a neighboring gene [34]. To rectify this, we made a clean knock-out of the stathmin locus by homologous recombination, removing most exons encoding the conserved part of the protein, but not affecting neighboring genes (Fig. 3A). Homozygous mutant animals were viable, classifying stathmin as a non-essential gene, but male sterile and with reduced female fertility and movement disorders. The defects were rescued by transgenic expression of a stathmin cDNA. The homozygous stathmin mutant females (stai^KO^) allowed us to determine whether Stathmin overall had a role in border cell migration, whereas genetic mosaics in which border cell clusters were devoid of stathmin but the rest of the tissue had a wild type allele (stai^KO^ clone) allowed us to determine the role in the migrating cells themselves. This comparison was relevant, as Stathmin is also expressed in the germline cells. Gross microtubule organization appeared normal in stathmin mutants (data not shown), consistent with stathmin being a non-essential regulator. For initial phenotypic analysis of border cell migration, we scored cluster position in fixed samples. A significant number of egg chambers showed migration delays, for both mutant situations (Fig. 3B). Analysis of late stage samples showed almost all had completed migration. Thus Stathmin has a role in border cell migration, but it is not essential for the process.

To better characterize the role of Stathmin, we performed live imaging experiments similar to those described for drug treated samples. Stathmin mutant egg chambers showed normal rate of initiation of border cell migration (Fig. 3C). Once clusters had initiated migration, however, there was a significant reduction of net migration speed (Fig. 3D). This defect was due to stathmin, as it could be rescued by transgenic expression of a stathmin cDNA (Fig. 3D). Border cell mutant clones in a heterozygous background showed a different effect: inefficient initiation of migration (Fig. 3C), but normal migration of the cluster once migration had properly initiated (Fig. 3D). Again, the defect was due to stathmin, as it could be rescued by transgenic expression of a stathmin cDNA (Fig. 3C). Thus the delays in migration at a specific stage in both fully mutant egg chambers and border cell mutant clones appeared to reflect two roles, one at initiation of migration, where stathmin acts in border cells, and another during migration where stathmin likely functions in the germ-line. The absence of initiation delay in mutant egg chambers was puzzling but indicates that effects on border cells and substrate are not simply additive. During migration, the absence of stathmin in border cells did not grossly change the frequency or direction of extensions, whereas fully mutant egg chambers did display altered extension profiles (Fig. 3E). Analysis of movies from stathmin mutant border cell clones revealed another phenotype: migrating border cell cluster often retained an attachment to the anterior end of the egg chamber for a long period (Fig. 3F), indicating that one border cell was still attached to a non-border cell from the epithelium (both are GFP positive and mutant in Fig. 3F). These “attached” clusters do migrate toward the oocyte, but slower than free ones. Attached clusters occur in control genotypes at a lower frequency but such clusters were excluded from the systematic migration analysis presented in Fig. 3D and E. Overall, the effects of stathmin mutants confirm that regulated microtubules are important both in the migrating cells and in the germ-line for invasive movement and that the roles are different in these two cell types.

### A Screen of Genes Encoding Microtubule Interacting Proteins Implicate Lis-1, NudE and Dynein in Migration

**Figure 4:**
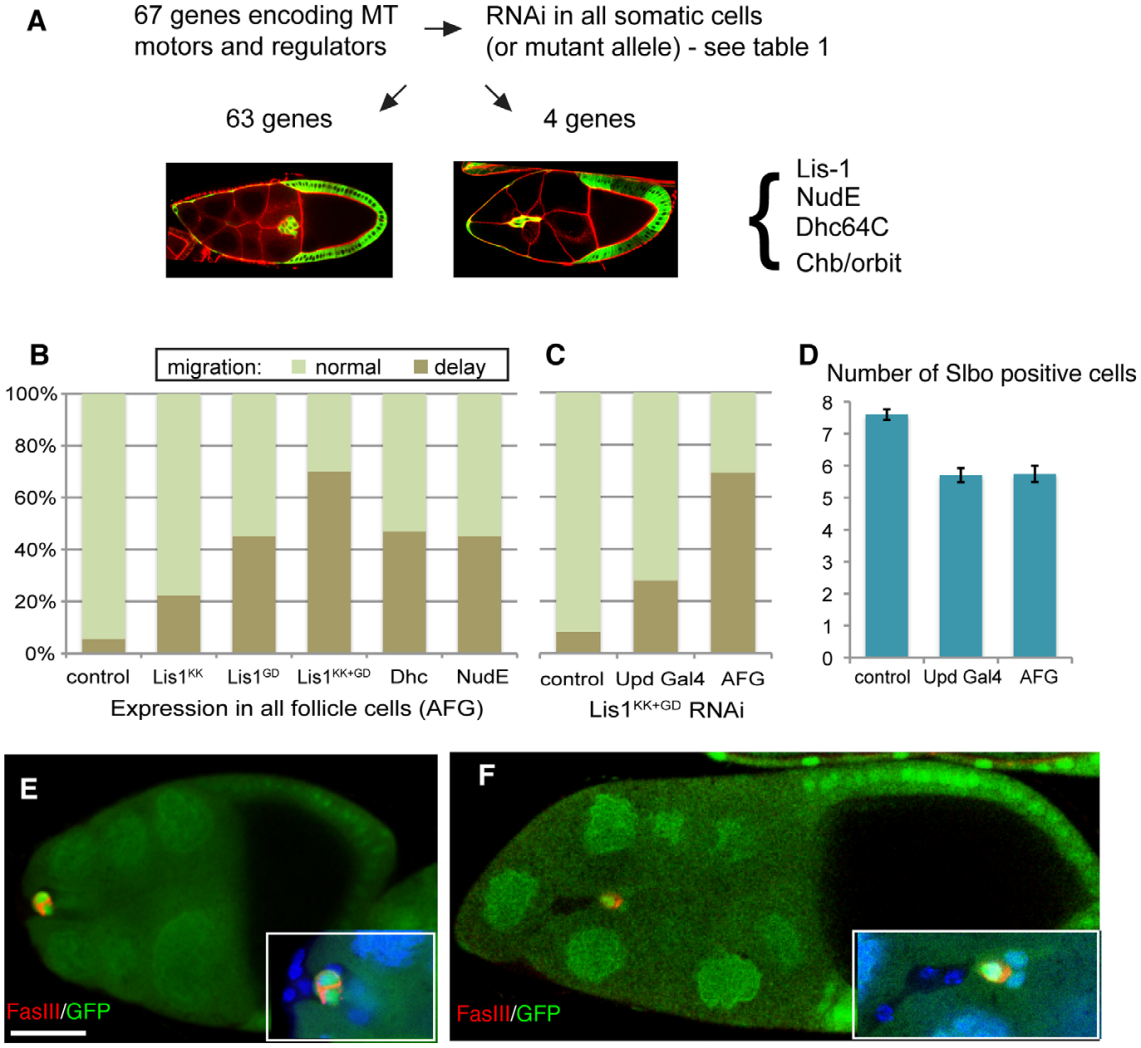
A screen of microtubule regulators and effectors implicates Lis-1 and Dynein. (A) Schematic of screen to identify microtubule regulators important in border cells. (B) Quantification of border cell migration status for mid/late stage 9 egg chambers with indicated RNAi knock-down in all follicle cells. Genotypes: hsFLP/+ AFG,10×GFP/+ and following RNAi lines: lis-1KK^108813^, lis-1GD^1480^, Dhc64C-TripJF^03177^, NudE-GD^29788^. All are significantly different from control (P<0.001 except for Lis-1KK P<0.05). (C) Quantification of border cell migration status for mid/late stage 9 egg chambers with Lis-1 knock-down in polar cells (UpdGal4) versus full cluster (AFG). Genotypes lis-1^KK108813^/+; lis-1^GD1480^/+ and no Gal4, Upd-Gal4 or AFG as in B. Difference between UpdGal4 and AFG is significant (P<0.0001). (D) Border cell specification effects. Number of Slbo positive cells per border cell cluster in stage 9 or 10 egg chambers; genotypes as in C. (E) Lack of migration in a clone of lis-1G^10^.^14^ mutant cells (marked by absence of GFP, green) in a late stage 9 egg chamber. All migratory border cells are mutant, but the polar cells, marked by FasIII (red) staining between them, are not. Genotype hsFLP/+; FRT^G13^,Lis-1^G10^.^14^/FRT^G13^,ubiquitinGFP. (F) Severely defective migration in a clone of Dhc64C^4−19^ mutant cells (marked by absence of GFP, green); stage 10 egg chamber. Most border cells are mutant and are in the rear; the polar cells marked by FasIII (red) staining between them, are not mutant. Genotype hsFLP/+; Dhc64C^4−19^,FRT^2A^/FRT^2A^,ubiquitnGFP. Scale bar: 30 µm. Expanded view (3×) in bottom corner of E and F also shows DAPI (blue) to mark all nuclei.

The modest effects of microtubule disruptions in border cells observed so far could indicate that the microtubule cytoskeleton only plays a minor role in these cells or that the perturbations were incomplete and the key genetic regulators had not been found. To identify additional regulators and effectors/motors of the microtubule cytoskeleton acting in the migrating cells themselves, we performed a systematic screen of a large set of genes encoding these functions (Fig. 4A and table S1). We analyzed loss-of-function mutant alleles or transgenic RNAi lines (usually 2 per gene) with expression turned on in all follicle cells using actin-Gal4 with a flipout cassette (AFG). AFG was used as previous experiments had shown us that border cell-specific expression of RNAi with Slbo-Gal4 only induced overt phenotypes for a subset of genes known to be important for border cell migration, with protein levels decreasing significantly only after migration was well underway. Screening 67 different genes in this manner only yielded 4 causing delays in border cell migration. Border cell clones of a hypomorphic allele of Chb (also called orbit, Mast or CLASP), encoding a microtubule plus-end tracking protein, showed mild delays in migration. Null mutant border cell clones could not be obtained and the RNAi lines tested had no effect so this gene was not pursued further. Depletion of three other genes by RNAi caused migration delays, namely Lis-1, Dynein (DHC64C) and NudE (Fig. 4B). Lis-1 and NudE work together with the microtubule minus end-directed motor Dynein to perform load-bearing transport [35], which is important for movement of nuclei, to organize the microtubule cytoskeleton and for cell migration in several contexts [11], [36]. It was notable how few genes were found to have a role in border cells by this approach. Complete gene deletion was generally not examined and the list of genes could be expanded, so some genes may have been missed. However, observing detectable effects from RNAi directed against each of these 3 genes encoding cooperating proteins suggests a reliable overall efficiency. This, in turn, supports the notion that only a few microtubule regulators and motors have uniquely required functions in border cells.

It has recently been shown that Dynein, Lis-1 and NudE function together in polar cells to allow efficient, polarized production and secretion of Unpaired [27]. As Unpaired is required for specification of migratory border cells through the activation of the JAK/STAT pathway in border cells, this could in principle explain the observed migration delays. However, when the strongest combination of Lis-1 RNAi was expressed in polar cells using Upd-Gal4, the migration defect was much milder than that seen with expression in all follicle cells (Fig. 4D). This despite the fact that these two treatments produced an equivalent decrease in specification of migratory border cells, as measured by number of border cells expressing Slbo at stage 9 or 10 (Fig. 4D). This suggested that Lis-1, Dynein and NudE have a specific function in migratory border cells, in addition to their role in polar cells. To test this more rigorously, we analyzed genetic mosaics. We first analyzed the rare cases (4) where the AFG-driven expression of Lis-1 RNAi had been turned on in border cells but not polar cells. These clusters were all delayed. We then tested loss-of-function alleles of Lis-1 and DHC64. We looked for clones in which several migratory border cells, but none of the polar cells, were mutant. Such clones were rare (2 for Lis-1, 2 for DHC) but in each case severe border cell migration delays were observed (Fig. 4E, F). In addition, for clusters with border cells of different genotypes, the mutant cells were always in the back (Fig. 4F), a hallmark of mutations specifically affecting the migratory cells. As for the RNAi experiments, the effects of Lis-1 and DHC removal were similar, supporting that they work together in this context. We conclude that Lis-1, Dynein and NudE together perform a critical function in the migratory border cells and that RNAi-mediated gene reduction can mimic the mutant effects.

### The Lis-1 Complex is Critical for Initiation of Border Cell Migration and Cluster Organization

**Figure 5:**
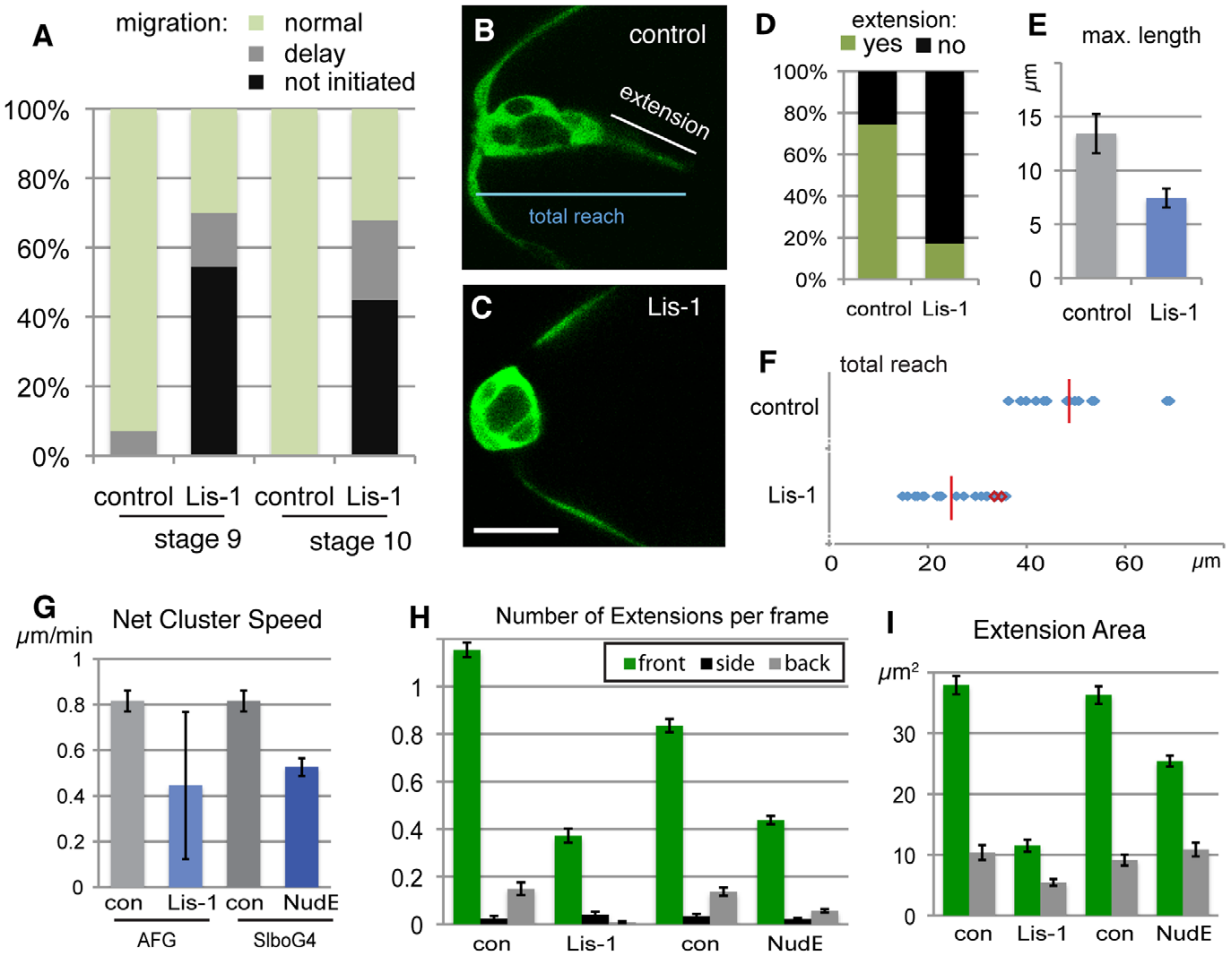
Lis-1 affects initiation of migration, and actual migration, of border cells. (A) Quantification of border cell migration status for stage 9 and stage 10 egg chambers. Genotypes hsFLP/+; AFG,10×GFP/+and hsFLP/+(control); hsFLP/+;lis-1^KK108813^/+;AFG,10×GFP/lis-1^GD1480^ (Lis-1). (B, C) Stills from movies of border cells (marked by GFP) at initiation stage (early stage 9) genotypes as in A. Scale bar: 20 µm. (D) Presence of forward extension, per frame, from movies as in B,C. (E) Maximum length of extensions manually identified from movies of early stage 9 clusters (n = 15, 25) as in B, C. (F) Total forward reach (see B) for control clusters at time of detachment from anterior end (n = 15); for Lis-1 RNAi clusters maximal forward reach in movie, only 3 (red) detach (n = 21) (G) Quantification of net speed from movies of migrating border cell clusters. Genotypes as in A as well as slboGal4,10×GFP/+ (control) and slboGal4,10×GFP/nudE^GD15226^ (NudE); n = 3 for Lis-1 (escapers) n = 13 for NudE and difference to control is significant (P<0.01). (H) Presence of cellular extensions from the migrating cluster (per movie frame), genotypes as in G. Differences to control are significant for both NudE and Lis-1 (P<0.0001). (I) Average area of front and back extensions from migrating clusters. genotypes as in F. Front extensions were significantly different from control for both Lis-1 and NudE (P<0.0001).

Strong reduction of Lis-1 expression had a severe effect on border cells. This was evident from analysis of fixed samples, where half of the egg chambers showed no migration had occurred (Fig. 5A). Similar defects were seen in stage 9 and in stage 10 egg chambers, when border cells normally migrate or have completed migration, respectively. Together, this suggested a block in initiation of migration. Live analysis of border cell clusters at the stage when migration should initiate showed Lis-1 knock-down clusters were more rounded than control (compare Fig. 5B and 5C). In wild type border cells clusters, we observed prominent extensions from the front cell before and at start of active cluster movement (Movie S5). All clusters analyzed showed this behavior. The invasive step did not necessarily occur upon formation of the first or longest forward extension, indicating this was not sufficient for initiating movement (Fig. S1). Instead, initiation was best correlated to the total forward reach of the cluster (see Fig. 5B), occurring when it was on average 49 µm and at least 36 µm (Fig. 5F). This is consistent with a long forward cell extension being important for initiation of migration by contributing to the full forward reach of the cluster. In Lis-1 knock-down movies (Movie S6), these early extensions were rarer (Fig. 5D) and did not reach the same size as in control movies (Fig. 5E). The maximum total reach of the Lis-1-depleted clusters ranged from 15 µm to 36 µm (Fig. 5F). In agreement with the fixed sample analysis, migration was rarely observed in these movies (3 of 21). Clusters that did initiate movement were 3 of the 5 most extended ones (red outlines in Fig. 5F), further supporting the importance of this feature for initiation of migration.

The escapers, the few Lis-1 knock-down clusters that initiated migration, allowed us to determine the effect of strong Lis-1 reduction on the migration process itself. The net movement of clusters was reduced to about half (Fig. 5G) with normal single cell speed (1.7 µm/min). As for the initiating clusters, the frequency and size of forward extensions was severely reduced (Fig. 5H–I). Our previous quantitative analysis of wild type clusters would suggest that a reduction of forward extensions as severe as that observed upon Lis-1 reduction would, on it own, result in the observed reduction in net forward cluster speed [23]. To determine whether the Lis-1 escaper clusters truly represented the effect of reducing of Lis-1 complex function in migrating border cells, we repeated the live analysis of border cells migration with NudE RNAi expressed under control of the border cell-specific Slbo-Gal4. As discussed, Slbo-Gal4 allows only relatively late knock-down of gene function but may cause partial loss-of-function effects if the gene product affected is not too stable. NudE RNAi with Slbo-Gal4 showed migration delays in fixed samples and was therefore used for live migration analysis. We observed very similar effects to those with Lis-1, both in terms of net migration speed (Fig. 5G) and presence of extensions (Fig. 5H), confirming that the phenotypes observed were indeed indicative of Lis-1/NudE complex function. Because the NudE knock-down was induced after border cell specification and only in migratory border cells, these effects also showed that the effect was cell autonomous (not due to polar cells) and that it was not an indirect effect of long-term depletion, as could have been the case with Lis-1 knock-down. We conclude that one function of the Lis-1/NudE complex in border cells is to allow formation of large cellular extensions; front cell extensions are, in turn, required for initiation of migration and allow more efficient forward movement of the cluster.

**Figure 6:**
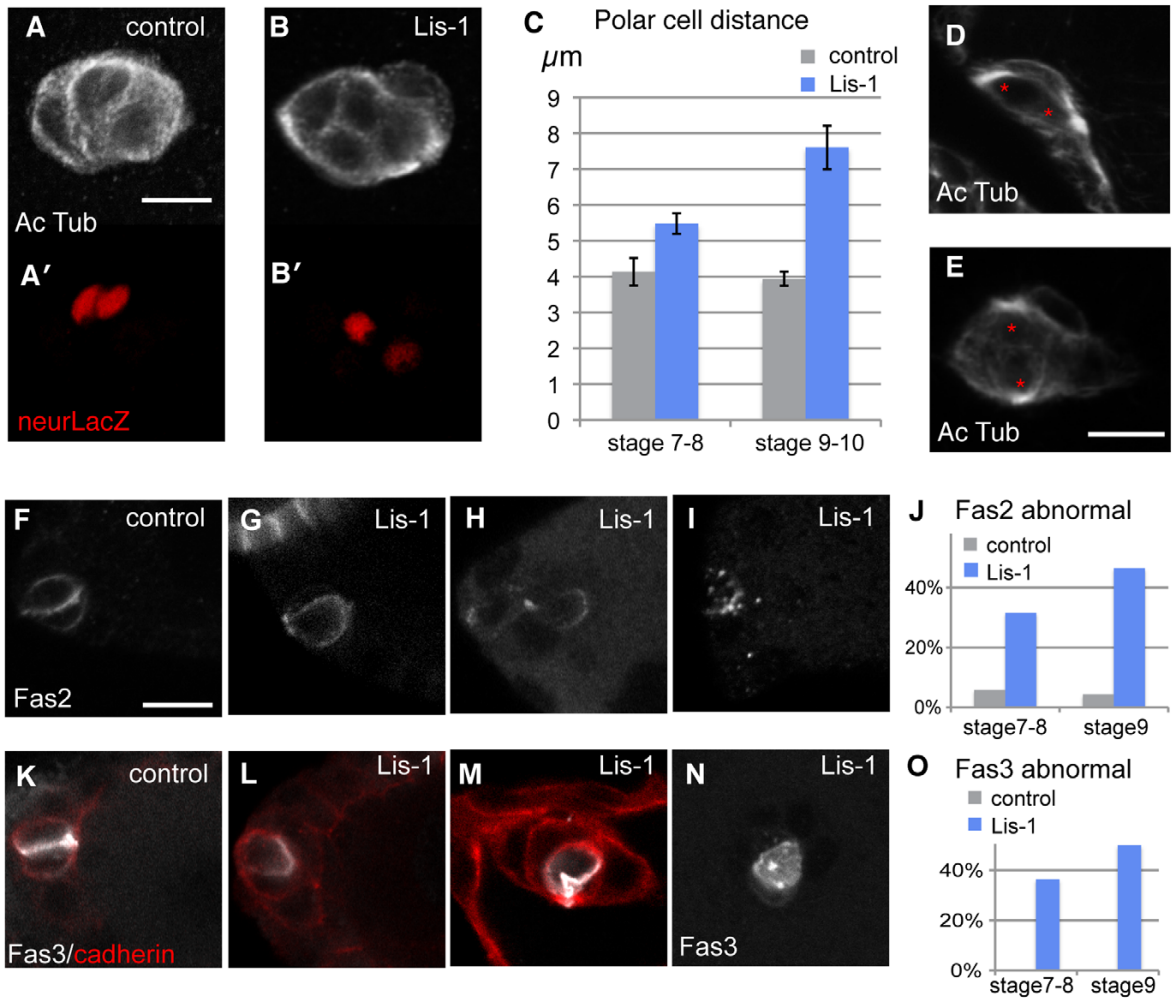
Lis-1 affects organization of the border cell cluster. (A–B) Migrating border cell clusters stained with anti-actylated tubulin (white) and anti-bgal (red) from lacZ in neur^A101^; genotypes: hsFLP/+;AFG/+;UAS-RFP,neur^A101^/+ (A) and hsFLP/+; lis-1^KK108813^/+;AFG/+;UAS-RFP, neur^A101^/lis-1^GD1480^ (C) Distance between 2 polar cells in a clusters as in A, B, measured between center of nuclei, in 3D. (D-E) Non-migrated (D) and migrating (E) Lis-1 depleted clusters show similar polar cell displacement. Note also the rotated MTOC-like structure in both. Anti-actylated tubulin (white); red asterisks indicate centers of polar cell nuclei, determined as in B; genotype as in A, B. (F) Normal stage 8 staining for Fas2 (white); (G-I) Examples of abnormal Fas2 localization in Lis-1 depleted clusters at stages 8 and 9. (J) Quantification of Fas2 localization at stage 7–8 and stage 9; n = 17–28. (K) Normal stage 8 staining for Fas3 (white) and Cadherin (red); (L-N) Examples of abnormal Fas3 localization in Lis-1 depleted clusters at stages 8 and 9. (O) quantification of Fas3 localization; n = 8–14. Genotypes for F- O: hsFLP/+; AFG,10×GFP/+ (control) and hsFLP/+;lis-1^KK108813^/+;AFG,10×GFP/lis-1^GD1480^ (Lis-1). Scale bars: 10 µm.

The strong effect of Lis-1 on initiation of border cell migration prompted us to analyze cellular effects of Lis-1 depletion in more detail. Cell polarity and associated regulators are important for migratory cells, including for border cells [37], [38]. Complete removal of Dynein disrupts apical/basal polarity in follicle cells [39]. Lis-1 mutant null clones displayed similar effects, but reduction of Lis-1 level by RNAi did not (Fig. S2). Similarly, microtubule polarity in the epithelial cells as revealed by live EB1-GFP tracing was also essentially normal in Lis-1 reduced cells (Fig. S2), indicating the residual amount of Lis-1 protein was generally sufficient to maintain simple epithelial cell polarity. However, in Lis-1-depleted border cell clusters, detection of stable microtubules revealed an altered microtubule cytoskeleton (compare Fig. 6A and 6B, top panels). The MTOC-like structures normally observed in the apical aspect of the adjacent polar cells were mostly lost or altered. Analysis of a nuclear polar cell marker showed that overall cluster organization was perturbed: the polar cells were no longer close together (see lower panels of Fig. 6A and 6B). Polar cell displacement was a progressive defect, more severe in clusters of stage 9 or 10 egg chambers than at earlier stage (Fig. 6C). However, it was observed both for clusters unable to initiate migration (Fig. 6D) and those that did (Fig. 6E). Large distance between the MTOC-like structures was frequently seen as well (Fig. 6D and 6E). A minority of clusters also showed abnormal number of polar cell nuclei; such clusters were not included in the quantitation. Thus reduction of Lis-1 caused mis-organization of microtubules that could be most easily observed in polar cells and apparent detachment of these normally very tightly associated cells.

To understand how the cluster mis-organization might arise, we considered what normally happens when the cluster is being formed. Cells of the cluster must rearrange upon initiation of migration, in the transition from epithelium to cluster topology (stages 8 to 9, see Fig. 1E). Live imaging of this stage showed actin-dependent rotational or jostling movement of clusters prior to invasion, starting from when clusters rounded up (movie S7 and Fig. S3). Changes in adhesion between the polar cells and the migratory cells might be expected to permit polar cell displacement during this and subsequently phases, whether invasion takes place or not, and in a progressive manner. Fasciclin 2 (Fas2, Fig. 6F), similar to mammalian NCAM2, and Fasciclin 3 (Fas3, Fig. 6K), another homophilic cell adhesion molecule, normally become enriched in polar cells. Fas3 localizes almost exclusively to the polar cell - polar cell interface and Fas2 in a slightly more complex pattern (see also [40]. Their localization was abnormal in many Lis-1 depleted clusters (Fig. 6G–I and Fig. 6L–N). The altered localization could contribute to polar cell displacement or might be a consequence of it. Analysis of Lis-1-depleted clusters at different stages showed largely abnormal protein distributions even before the onset of rounded cluster formation (stage 7–8 in Fig. 6J and Fig 6O). Thus, altered distribution of adhesion complexes appears to precede polar cell displacement. This is at least consistent with changes in cell-cell adhesion being a direct result of Lis-1 depletion (or of the mis-organized microtubule cytoskeleton produced by Lis-1 depletion) and cellular organization of the cluster being altered as a result of changes in adhesion.

## Discussion

Initiation of invasive migration by the border cell cluster is linked to the formation of a robust forward-directed extension from the front cell [25] and this study). During migration such extensions promote more efficient net forward movement of the cluster [23]. The formation and growth of this prominent structure was severely affected by Lis-1 and NudE depletion, which can explain the observed migration effects. However, the front cell extension was not severely perturbed by cell-autonomous reductions in microtubules, indicating that the microtubule cytoskeleton as such might not be essential for the structure. If Lis-1, NudE and Dynein were required to produce load-bearing movement of the front cell nucleus or cell content, the expectation is that the microtubule cytoskeleton would be required as well. Instead, the misorganized microtubule cytoskeleton caused by lack of Lis-1 or NudE could be the cause of the observed defects and might be more disruptive for polarized migratory cells than loss of microtubules altogether. We cannot formally rule out a microtubule-independent function of Lis-1 and NudE, but given the established interactions with Dynein and the phenotype of Dynein depletion as well as microtubule alterations observed here, this seems unlikely. The role of Lis-1 and Dynein in polar cells that indirectly affects border cell specification [27] may also contribute to the observed effects but cannot be the main driver as discussed above. Finally, in migrating border cell clusters, microtubule depletion stabilized outwards extensions from rear cells (back extensions), while having minimal effect on the robust front cell extensions. One way to reconcile these data and Lis1 mutant phenotypes would be that microtubules can destabilize susceptible cellular extensions and misorganized microtubules will cause this to occur at the wrong place, namely in the front cell.

Comparison of tissue-effects with cell-autonomous effects of loss of microtubules or loss of Stathmin indicated that microtubules play a role in both the migrating cells and the surrounding substrate cells. For example, the reduced cell motility observed immediately upon nocodazole treatment appears to be due to loss of microtubules in the substrate cells (or both cell types). The bias for microtubule growth in border cells was also different from that observed in many other migratory cells, which have a prominent MTOC [2], [7]. In border cells, the overall bias likely reflects that these cells were polarized epithelial follicle cells before reorganizing into a migratory cluster, both without an MTOC. But how should the bias be reconciled with effects in other cell migration systems? One additional difference is the migration substrate: for border cells it is cellular, for many well-studied migratory cells, it is ECM or culture dish surface. One possible explanation for both autonomous and non-autonomous effects of microtubule disruption is that the cell-cell adhesions responsible for generating protrusions and traction are affected. In the case of border cells migrating on nurse cells, this appears to be mediated by DE-cadherin mediated adhesion between the two cell types [19], [20]. Microtubules have in other contexts been shown to regulate cadherin-dependent adhesion or adherens junctions, and vice-versa [41], [42] and we did observe a genetic interaction with DE-cadherin. Other phenotypes observed in this study, namely prolonged attachment of border cells to their neighboring follicle cells in stathmin mutant clones and reorganization of cells and redistribution of adhesion molecules following Lis-1 depletion, also imply altered cell-cell adhesion - within the cluster or with neighboring somatic cells. As the adhesion required for cell-on-cell migration is different from that required for migration in cell culture conditions, this might also relate to the differences in microtubule polarity. Overall, it is tempting to speculate that spatial regulation of cell-cell adhesion is a key role of the microtubule cytoskeleton in this system.

It is interesting to compare neuronal migration in the brain to border cell migration as both represent cell-on-cell migration in crowded environments and as Lis-1 appears to have important roles in both. The effects of Lis-1 depletion on neuronal migration in the brain has been studied using knock-down approaches, which depending on the approach can target a few or all cells [43], [44] as well as in mutant animals [45]. These studies show a prominent effect of Lis-1 depletion, and of Dynein depletion, on cell and nuclear movement as well as an effect on axonal extensions, which was also observed in culture [46], but not on all extensions. Importantly, strong defects in switching to the migratory state were also observed [43]. For neuronal progenitors, this involves a change from one differentiated cell state and shape to another (multipolar to bipolar), just as border cells re-organize to become migratory (epithelial to cluster). Such transitions may be particularly sensitive to Lis-1 levels. Recently, application of a new genetic mosaic technique allowed detailed analysis of individual neurons lacking or heterozygous for Lis-1 or Ndel1 (one of two NudE-related proteins in mammals) and comparison to normal sibling cells [47]. This study revealed an unexpected degree of non-cell autonomy of phenotypic effects for both Lis-1 and Ndel1. It was speculated that the non-autonomous functions were community effects (“piggy-backing”), an effect that can be observed for cells migration collectively [15], or cell-cell signaling effects. It is also possible, that as suggested for microtubule effects in this study, that requirements for Lis-1/Ndel1 might be liked to cell-cell adhesion: effects in the migrating cell as well as in the substrate cells. There are obviously significant differences between neuronal and border cell migration. For example, migrating neurons have a prominent centrosome, which may act as MTOC, and microtubule polarity is predominantly plus end outwards [44], [48]. But the common features of cell-on-cell migration by different cell types may be informative as well and are clearly worth further investigation.

## Materials and Methods

### Drosophila Strains and Genetics

Ubi-EB1-GFP (Poly-ubiqutin promoter driven EB1-GFP [49] was used for all EB1-GFP tracking experiments. Other flies used: Tubulin-GFP [50], UAS-spastin-EGFP [30], UAS-10×GFP [23]; neur^A101^ was used to mark polar cells. Mutants and RNAi lines were obtained from Bloomington and Vienna stock centers (details in table S1 and Flybase (http://flybase.org/) for additional information. Genotypes used in different experiments are given in the legends to each figure.

For RNAi expression in the screen, hs-FLP was combined with RNAi lines and crossed to actin-flip-out Gal4 (AFG) with a slbo-lacZ transgene on X. Larvae were heat shocked for 30 minutes and ovaries from 1–2 day old female were dissected, except for few lines that early expression resulted in lethality (Msps, ssp4, spastin, tektin-C, sw, unc-104, ctp and klp64DLis-1, Dhc64C), for which heat shock was given to adult females instead and ovaries were dissected up to 4 days post heat shock. Xgal staining was done according to standard procedures and >100 stage 10 egg chambers were scored. If delays were observed, the RNAi line was retested as above but with AGF, UAS-10×GFP and confocal analysis, scoring at both stage 9 and 10.

For clonal analysis, stai^KO^ mutants were recombined with FRT40 and clones were induced by heat shocking larvae of the genotype hsFLP/+; stai^KO^, FRT40,42,/FRT40,Gal80; slboGal4,UAS-10×GFP/+; completely GFP positive clusters were analyzed. FRT40,42 was used to induce empty clones as control. For Lis-1 clones, FRT^G13^,Lis-1^G10^.^14^ (BL8773) was used and adult flies with genotype hsFLP/+; FRT^G13^,Lis-1^G10^.^14^/FRT^G13^,ubiquitinGFP were heat shocked at 37 degree for 30 minutes and ovaries were dissected 3–4 days later. For Dhc64C clones, Dhc64C^4–19^,FRT^2A^ (BL23863 ) was used and adult females with genotype hsFLP/+;Dhc64C^4–19^,FRT^2A^/FRT^2A^,ubiquitinGFP were heated shocked. Dynein mutant clones were also generated in e22cgal4, UAS-FLP/+; Dhc^4–19^, FRT^2A^/UbiGFP, FRT^2A^ females, which were kept at 30°C before dissection. There was not obvious difference in clone frequency or border cell migration phenotype achieved between the two methods.

### Generating stai^KO^ Mutant and Rescue Flies

The *stai^KO^* mutant was generated using homologous recombination-based ends-out gene targeting [51] and the vector pW25. The stai^KO^ knock-out vector included two homology arms with about 3.5 kb upstream (primers: 5′-ttgcggccgctctattatggcgggttatgc-3′ and 5′-ttgcggccgcaggaggaaggaaagcaaagg-3′) and downstream (primers 5′- TTGGCGCGCCGCATGGCCAAAAGTTTTCAT-3′ and: 5′-TTGGCGCGCCCTACGAGAACGCAGTGGTCA-3′ of the region to be removed. The deleted region starts from nucleotide sequence encoding exon 6 and finishes at exon8), resulting in the disruption of translation from Thr55 as in stathmin A. 14 independent lines were verified to have the correct event by PCR with 5′-aagaacgttagcgtcgagga-3′ and 5′-ctcctttaggcgatccaaca-3′ Several staiKO lines were lethal but not lethal over a small deficiency for the region L27 (Borghese et al., 2006). Viable transheterozygous combinations of staiKO lines were generally used for the phenotypic analysis.

To make pCasper-attB-tubulin-stathmin, the tubulin promotor from pCasper-tubulin was cloned into pAttB [52] followed by the cDNA of stathmin A subcloned from pBS-stai [17]. To make pUAST-attB-stathmin rescue constructs, the cDNA was subcloned to pUAST-AttB [52]. Transgenic flies were made by PhiC31 integrase-mediated transgenesis systems at targeted insertion site 86 Fb.

### Live Imaging and Data Analysis

Egg chambers were dissected and cultured as described previously [22]. Images were acquired by confocal microscopy (SP5, Leica), and for all migration analysis, movies were assembled and analyzed using customized macros as described previously [23]. For imaging border cell migration, Z sections 3 µm apart covering the entire border cell cluster were captured at between 30 sec–120 sec intervals. Analysis was done from movies covering up to 50% migration path with a minimum duration of 20 min. Net cluster migration was based on start position and end position of the cluster center.

For tracking EB1-GFP dots, generally a single section was captured at 4× zoom with 0.6 seconds per frame (or slightly slower) time resolution. The Image J plugin MTrackJ (http://www.imagescience.org/meijering/software/mtrackj/) was used for tracking; this was converted into a vector and its angle measured relative to forward direction to the oocyte and separated into forward (0–45° and 315–360°), backwards (135–225°) and sideways (the rest).

Drug treatment: For nocodazole [Sigma], various concentration gradients were tested and 2 µM was shown not to disrupt the overall development of the egg chamber. For taxol [Sigma], a final concentration of 2 µM was used. Vehicle DMSO [Sigma] was used as control. For migration analysis, imaging was set up immediately after dissection with an average lag period about 10 minutes.

### Immunostaining and Analysis

Ovaries were dissected in Schneider medium (Gibco) with 0.5 µM insulin (Sigma) and fixed in 4% para-formaldehyde [Electron Microscopy Sciences] for 20 minutes; antibody staining was done using standard procedures, except for 30 minutes incubation in PBS+1%tritonX-100 for anti-tubulin staining. The following primary antibodies were used: mouse anti-alpha-tubulin (1∶5000; DM1A, Sigma); mouse anti-actylated-tubulin (1/1000; T7451, Sigma); rat anti-Slbo (1/500); mouse anti-FasII (1/100; 1D4, Developmental Studies Hybridoma Bank (DSHB); mouse anti-FasIII (1/100; 7G10, DSHB); rat anti-DE-cadherin (1∶100, DCAD2, DHSB), rabbit anti-aPKC (1/2000, sc-216, Santa CRuz), rabbit anti-beta Gal (1/1000, Cappel). Secondary antibodies used were Rhodamine (TRITC), Cy5 or Dylight-649 conjugated (Jackson ImmunoResearch). Alexa Fluor 546-Phallodin [Molecular Probes] was used for visualized F-actin and DAPI was used to visualize nuclei. Images were acquired with a Zeiss, LSM700 confocal with 40× oil-immersion objective. Usually, Z-sections of 2 µm were taken to cover the entire border cell clusters.

### Statistics

All statistic analysis was done by a two-tailed student t test except for the comparisons of percentage of phenotype in which the Fisher’s exact test was used (http://www.graphpad.com/quickcalcs/contingency1.cfm).

## Supporting Information

Figure S1 **Front extensions at the onset of migration.** Tracking forwards extensions from control (*slbo-Gal4,UAS-10xGFP/+*) border cell clusters at initiation of migration. Two examples of the 17 movies analyzed are shown with size of front extension determined automatically over time as for migrating clusters in (Poukkula et al., 2010). Time-point for cluster detachment from the anterior is indicated. Type A are clusters where detachment happens after the first long extension (green arrow); type B detachment happens after a subsequent long extension (blue arrow). Below are plots of size of front extension or of total forward reach at time of detachment; the X-axis indicates the length of oocyte along anterior-posterior axis for the same egg chamber as a sensitive indication of developmental stage (within early-mid stage 9).
(TIF)

Figure S2 **Assaying apical-basal polarity upon Lis1 disruption.** (A–B) Follicle cells from stage 9 egg chambers stained with aPKC (white) in (A) Lis-1^G10^.^14^ clones marked with absence of GFP (green) and (B) Lis-1 expressing cells marked positively with GFP. Scale bar: 5 µm. (C) Quantifications of directions of tracked EB1-GFP comets in control and Lis-1RNAi expressing follicle cells. Genotypes: *hsFLP/+; AFG/+, ubiqutin-EB1-GFP, UAS-RFP/+* (control) and *hsFLP/+;AFG/Lis-1^KK106777^, ubiqutin-EB1-GFP,UAS-RFP/Lis-1^GD6212^* (Lis-1). 16 tracks from control and 31 tracks from Lis-1 RNAi were analyzed.
(TIF)

Figure S3 **Analysis of early rotation movement.** Rotating movement in border cell clusters at initiation of migration (*slbo-Gal4,UAS-10xGFP/+*), compared to posterior follicle cells at the same stage and border cells in egg chambers treated with 1µM of cytochalasin D (n = 7–9 clusters, two cells tracked per cluster). The angle from cluster center to nucleus is tracked. The baseline “movement” may mostly be intracellular nuclear movement and manual tracking inaccuracies. See movie S7 for corresponding border cell movies.
(TIF)

Table S1 **Full list of genes analyzed in the screen.**
(DOC)

Movie S1 **Live imaging of EB1 dynamics from egg chambers expressing** ***ubiqutin-EB1-GFP*** **.** A single confocal section of a border cell cluster initiating migration (not yet detached) is shown. Time interval between frames was 0.6 sec. A section of this movie was used for the stills in Fig. 1G
(AVI)

Movie S2 **Movie showing early phase of border cell migration.** All confocal sections were taken and projected in the GFP channel (green in upper panel and white in bottom panel). Projected GFP channel images were overlaid with the central section of the red channel of FM4-64 (membrane dye). Only the anterior part of the egg chamber is shown and the oocyte is off the image to the right; timestamp in minutes; the genotype is *slboGal4, UAS-10×GFP/+* (control).
(AVI)

Movie S3 **Movie showing early phase of border cell migration in an egg chamber treated with 2** **µM nocodazole.** Images and genotype as described for movie S2.
(AVI)

Movie S4 **Movie showing early phase of border cell migration in egg chamber treated with 1** **µM of cytochalasinD.** Images and genotype as described for movie S2.
(AVI)

Movie S5 **Early stage 9 egg chamber showing control border cell cluster initiating movement and detaching with a prominent extension from the front cell.** One section of the GFP channel (green at top, white at bottom) and red channel (FM4-64 membrane dye) slicing through the middle of the cluster is shown; polar cells show less bright GFP and nuclei very little GFP. The anterior is to the left, and the oocyte is off the image on the right. Genotype is *slbo-Gal4,UAS-10xGFP/+.*
(AVI)

Movie S6 **Early stage 9 egg chamber showing Lis-1 RNAi expressing border cells at the stage when clusters should be initiating movement and detaching.** One section of the GFP channel slicing through the middle of the cluster is shown; polar cells show less bright GFP. The anterior is to the left, and the oocyte is off the image on the right. Genotype is *hsFLP/+;lis-1^KK108813^/+;AFG,10xGFP/lis-1^GD1480^*
(AVI)

Movie S7 **Early stage 9 egg chamber showing control border cell cluster rotating, “jostling” movement prior to initiating movement.** Imaging and genotype as for movie S5.
(AVI)
