# Supplementary figures and images for: Microtubules and Lis-1/NudE/Dynein Regulate Invasive Cell-on-Cell Migration in Drosophila
Source: PLoS One. 2012 Jul 13;7(7):e40632. doi: 10.1371/journal.pone.0040632 (PMC3396602; doi:10.1371/journal.pone.0040632)

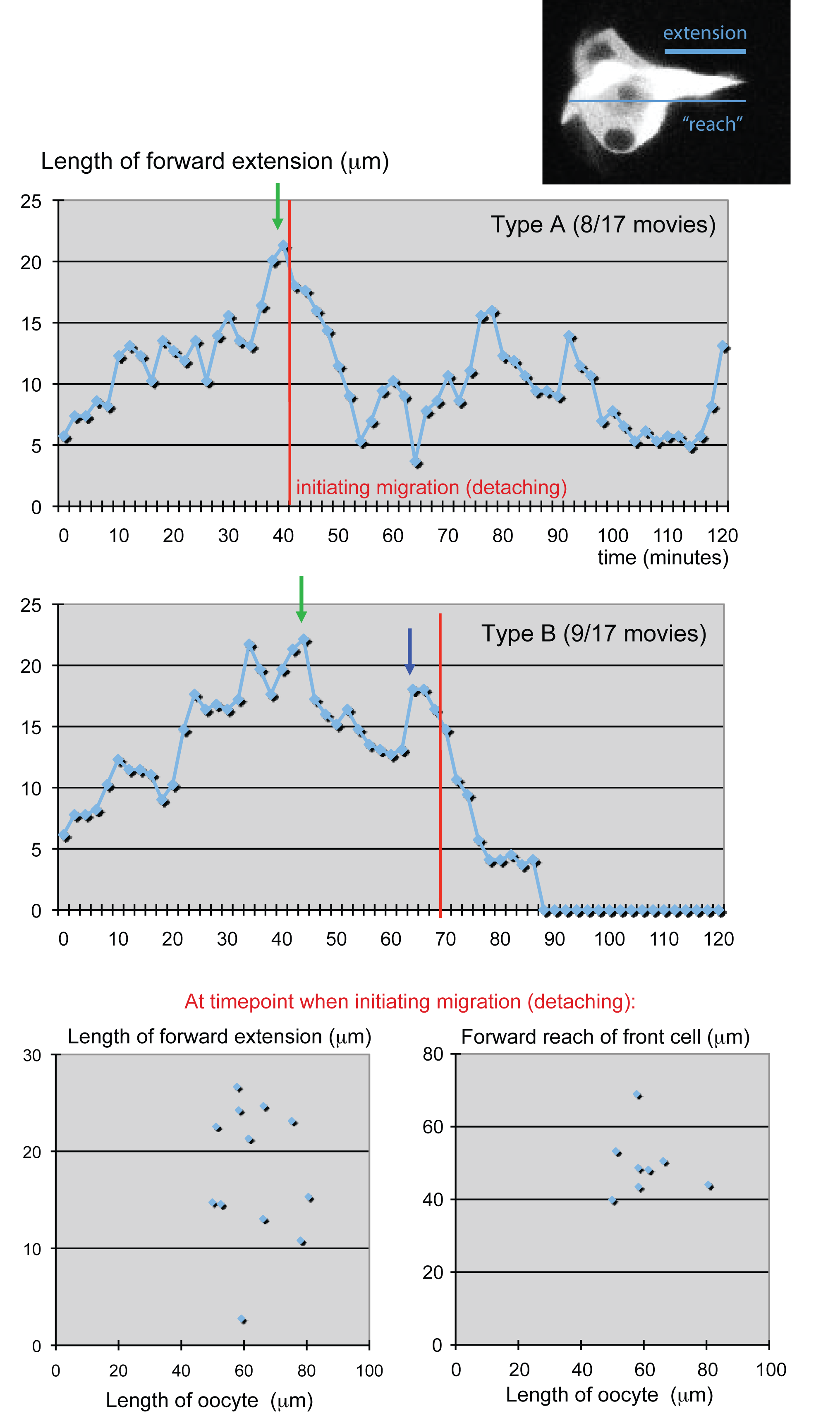

Supplement: Figure S1 — Front extensions at the onset of migration. Tracking forwards extensions from control (slbo-Gal4,UAS-10xGFP/+) border cell clusters at initiation of migration. Two examples of the 17 movies analyzed are shown with size of front extension determined automatically over time as for migrating clusters in (Poukkula et al., 2010). Time-point for cluster detachment from the anterior is indicated. Type A are clusters where detachment happens after the first long extension (green arrow); type B detachment happens after a subsequent long extension (blue arrow). Below are plots of size of front extension or of total forward reach at time of detachment; the X-axis indicates the length of oocyte along anterior-posterior axis for the same egg chamber as a sensitive indication of developmental stage (within early-mid stage 9). (TIF) [file pone.0040632.s001.tif]

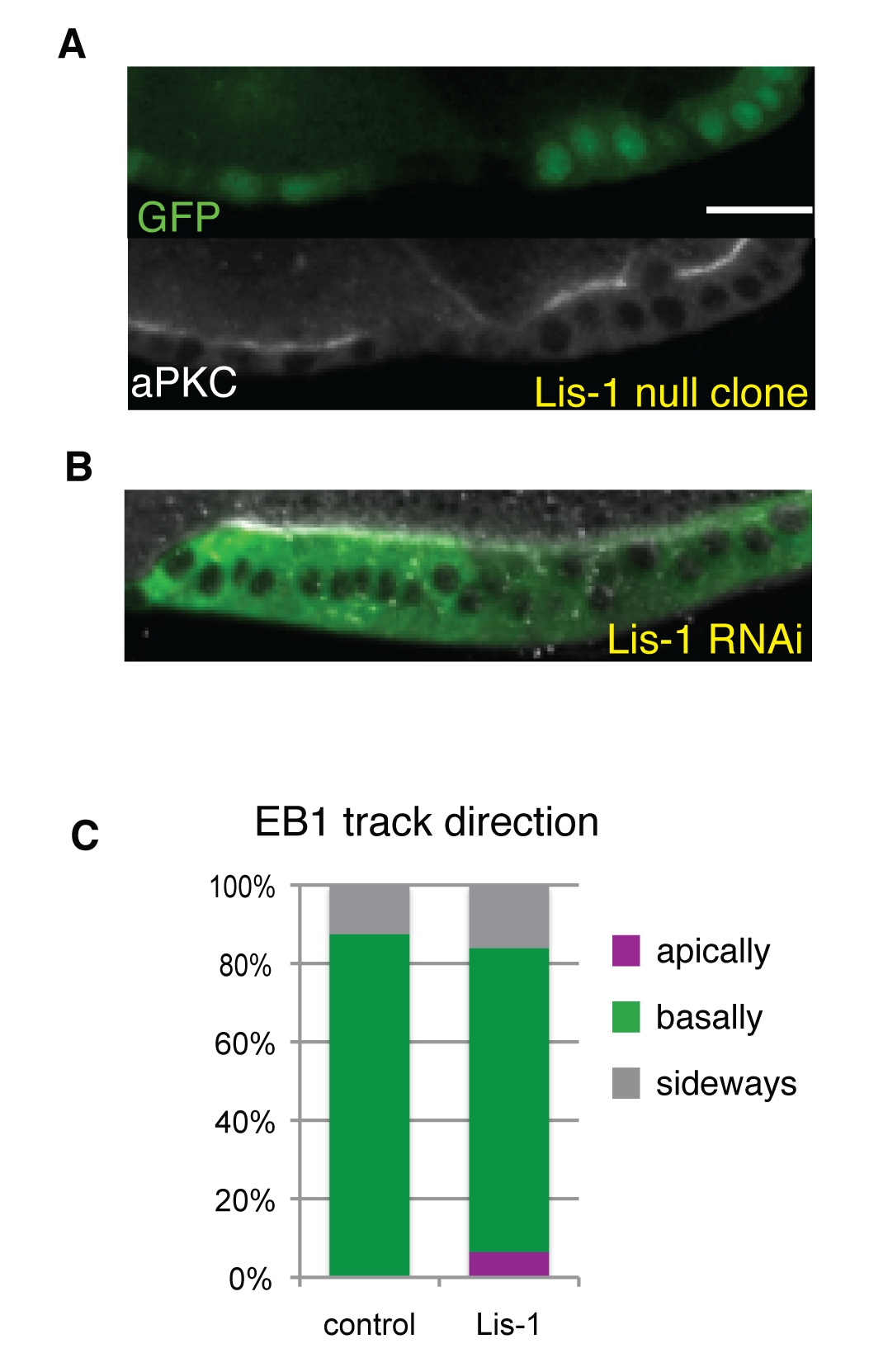

Supplement: Figure S2 — Assaying apical-basal polarity upon Lis1 disruption. (A–B) Follicle cells from stage 9 egg chambers stained with aPKC (white) in (A) Lis-1G10.14 clones marked with absence of GFP (green) and (B) Lis-1 expressing cells marked positively with GFP. Scale bar: 5 µm. (C) Quantifications of directions of tracked EB1-GFP comets in control and Lis-1RNAi expressing follicle cells. Genotypes: hsFLP/+; AFG/+, ubiqutin-EB1-GFP, UAS-RFP/+ (control) and hsFLP/+;AFG/Lis-1KK106777, ubiqutin-EB1-GFP,UAS-RFP/Lis-1GD6212 (Lis-1). 16 tracks from control and 31 tracks from Lis-1 RNAi were analyzed. (TIF) [file pone.0040632.s002.tif]

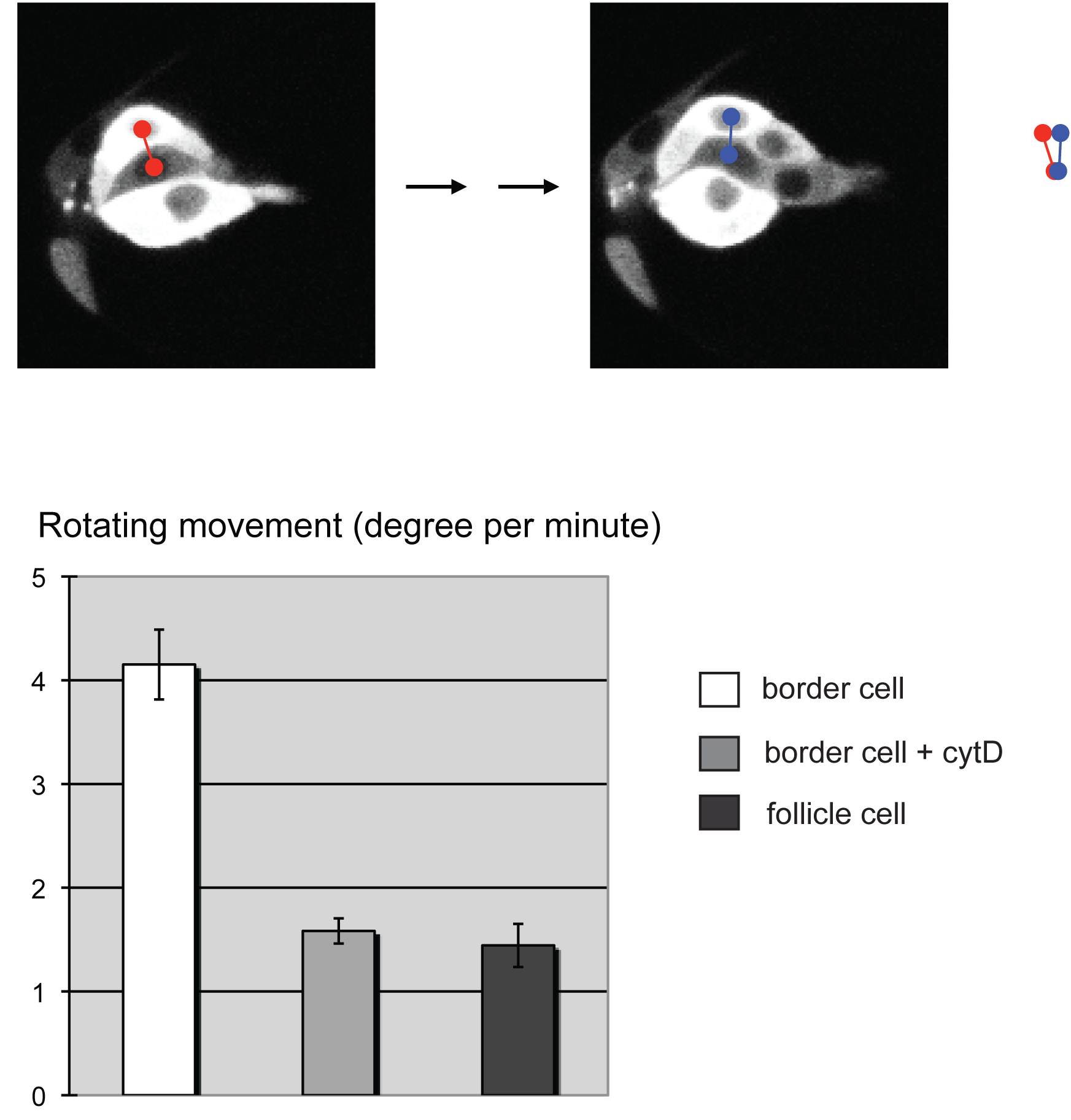

Supplement: Figure S3 — Analysis of early rotation movement. Rotating movement in border cell clusters at initiation of migration (slbo-Gal4,UAS-10xGFP/+), compared to posterior follicle cells at the same stage and border cells in egg chambers treated with 1µM of cytochalasin D (n = 7–9 clusters, two cells tracked per cluster). The angle from cluster center to nucleus is tracked. The baseline “movement” may mostly be intracellular nuclear movement and manual tracking inaccuracies. See movie S7 for corresponding border cell movies. (TIF) [file pone.0040632.s003.tif]
