# Supplementary material for: Microtubules and Lis-1/NudE/Dynein Regulate Invasive Cell-on-Cell Migration in Drosophila
Source: PLoS One. 2012 Jul 13;7(7):e40632. doi: 10.1371/journal.pone.0040632 (PMC3396602; doi:10.1371/journal.pone.0040632)
Supplement: Table S1 — Full list of genes analyzed in the screen. (DOC) [file pone.0040632.s004.doc]

Table 1: Full list of genes included in the screen

| Flybase ID | Gene | Function | Mutant alleles | RNAi lines | Migration |
| --- | --- | --- | --- | --- | --- |
| FBgn0032390 | dgt2 | MT nucleator |  | TRiP.HM04038 | normal |
| FBgn0034569 | dgt3 | MT nucleator |  | v103980 | normal |
| FBgn0026085 | dgt4 | MT nucleator |  | v108969 | normal |
| FBgn0033740 | dgt5 | MT nucleator |  | v26911 | normal |
| FBgn0039638 | dgt6 | MT nucleator |  | v108799 | normal |
| FBgn0026431 | Grip75 | MT nucleator |  | v106044 | normal |
| FBgn0032705 | Grip71 | MT nucleator |  | v100830 | normal |
| FBgn0026432 | Grip163 | MT nucleator |  | v108586 | normal |
| FBgn0026430 | Grip84 | MT nucleator |  | v105640 | normal |
| FBgn0001612 | Grip91 | MT nucleator |  | v2983 | normal |
| FBgn0027500 | D-Spd2 | MT nucleator |  | v101882 | normal |
| FBgn0036059 | NudE | minus end anchor |  | v29788 | **delay** |
| FBgn0027948 | Msps | MT aseembly promoter |  | v21982 | normal |
| FBgn0020503 | CLIP-190 | MT aseembly promoter |  | v107176 | normal |
| FBgn0004379 | Klp67A | MT disassembly promotor |  | v108852 | normal |
|  |  |  |  | Trip.JF02701 |  |
| FBgn0022085 | ssp4 | MT severing |  | v108927 | normal |
| FBgn0039141 | spastin | MT severing | spas^5.75^ | v108739 | normal |
| FBgn0040208 | katanin 60 | MT severing |  | v106487 | normal |
| FBgn0259108 | futsch | MT stabilizing | futsch^K68^ | v6973 | normal |
| FBgn0004378 | Klp61F | MT bundling |  | v109280 | normal |
| FBgn0021760 | Chb | MT stabilizing | chb^S068607^ | v108620 | **delay** |
|  |  |  |  | v26051 |  |
| FBgn0013733 | short stop | MT stabilizing | shot3 |  | normal |
| FBgn0040232 | cmet | MT stabilizing |  | v35081 | normal |
| FBgn0005316 | Mud | MT stabilizing | mud4 |  | normal |
| FBgn0026620 | D-TACC | MT stabilizing |  | v101439 | normal |
| FBgn0015754 | Lis-1 | MT stabilizing | lis-1^G10.14^ | v106777 | **delay** |
|  |  |  |  | v6216 |  |
| FBgn0028902 | tektin-A | MT stabilizing |  | v101714 | normal |
| FBgn0035638 | tektin-C | MT stabilizing |  | v100094 | normal |
| FBgn0029687 | DVAP33-1 | MT stabilizing | Vap-33-1^47^ | v100809 | normal |
| FBgn0041174 | D-VHL | MT stabilizing |  | v108920 | normal |
| FBgn0022959 | yps | MT aseembly promoter |  | v27473 | normal |
|  |  |  |  | v27472 |  |
| FBgn0000150 | awd | MT binding |  | v33198 | normal |
| FBgn0001108 | Glued | dynein binding |  | v3785 | normal |
| FBgn0001308 | khc | MT plus-end motor | khc^27^ | v44337 | normal |
| FBgn0000140 | asp | MT associated |  | v2911 | normal |
|  |  |  |  | v2910 |  |
| FBgn0000352 | cos | MT motor | cos^5^ | v108914 | normal |
| FBgn0011606 | Klp3A | MT plus-end motor | Klp3A^mei-352^ | v104682 | normal |
| FBgn0001612 | l(1)dd4 | MT minus-end binding |  | v104667 | normal |
|  |  |  |  | v2983 |  |
|  |  |  |  | Trip.JF01719 |  |
| FBgn0002924 | ncd | MT minus-end motor | ncd^D^ |  | normal |
| FBgn0004374 | neb | MT motor |  | v108138 | normal |
| FBgn0002948 | nod | MT plus-end binding | nod^a^ | v48148 | normal |
| FBgn0011692 | pav | MT motor |  | v46134 | normal |
| FBgn0003545 | sub | MT motor |  | v18754 | normal |
| FBgn0003654 | sw | MT motor |  | v101559 | normal |
| FBgn0034155 | unc-104 | MT motor |  | v23464 | normal |
|  |  |  |  | v47171 |  |
|  |  |  |  | v23465 |  |
| FBgn0014133 | bif | MT binding |  | v109722 | normal |
| FBgn0000256 | capu | MT binding | capu^EE^ | v34278 | normal |
| FBgn0032210 | CYLD | MT binding |  | v101414 | normal |
| FBgn0004167 | kst | MT binding |  | v37074 | normal |
|  |  |  |  | v37075 |  |
| FBgn0013726 | pnut | MT binding |  | Trip.JF02792 | normal |
| FBgn0003475 | spir | MT binding |  | v107335 | normal |
| FBgn0250788 | β Spectrin | MT binding |  | v42054 | normal |
| FBgn0004380 | klp64D | MT motor |  | v45373 | normal |
|  |  |  |  | v103358 |  |
| FBgn0086362 | spn-F | MT minus-end motor |  | v107850 | normal |
| FBgn0260991 | Incenp | MT binding |  | v101123 | normal |
|  |  |  |  | v17044 |  |
| FBgn0033687 | CG8407 | MT motor |  | v100696 | normal |
| FBgn0019968 | Khc-73 | MT motor |  | v105984 | normal |
| FBgn0035800 | CG7716 | MT nucleation |  | v104217 | normal |
| FBgn0004381 | Klp68D | MT motor |  | v101058 | normal |
| FBgn0038205 | Kif19A | MT motor |  | v106569 | normal |
| FBgn0030268 | Klp10A | MT motor |  | v41534 | normal |
| FBgn0026141 | Cdlc2 | MT motor |  | v42113 | normal |
| FBgn0011760 | ctp | MT motor |  | v109084 | normal |
| FBgn0034824 | Klp59C | MT motor |  | v109829 | normal |
| FBgn0052371 | CG32371 | MT binding |  | v106233 | normal |
| FBgn0261797 | Dhc64C | MT minus end motor | Dhc^4-19^ | Trip.JF03177 | **delay** |
| FBgn0001316 | klar | MT attachment | klar^1^ |  | normal |

Each gene was presented with its synonyms and functions. The unique Flybase identification number (ID) was shown on the left of each gene.

Mutant alleles include characterized amorph and hypomorph alleles or based on mutant phenotypes associated as reported in other system. RNAi lines starting with v are VDRC stock number; RNAi lines staring with Trip are TRiP line numbers from Trip Harvard Medical School. Mutants showing border cell migration delay are highlighted in bold.
